# Editorial: Polyamines and longevity - role of polyamine in plant survival

**DOI:** 10.3389/fpls.2023.1232386

**Published:** 2023-06-19

**Authors:** Luigi Parrotta, Ewa Sobieszczuk-Nowicka, Giampiero Cai

**Affiliations:** ^1^ Department of Biological, Geological and Environmental Sciences, University of Bologna, Bologna, Italy; ^2^ Department of Plant Physiology, Faculty of Biology, Adam Mickiewicz University in Poznań, Poznań, Poland; ^3^ Department of Life Sciences, University of Siena, Siena, Italy

**Keywords:** polyamines, abiotic and biotic stresses, nitrogen, senescence (leaf), cadmium stress

Polyamines (PAs) are organic polycations involved in stress and developmental processes in plants (Gentile et al., 2012; Gupta et al., 2013). PAs occur in cells and tissues in free (non-conjugated) or conjugated forms by binding to various molecules, including DNA and RNA, proteins, and membrane phospholipids, thus regulating various molecular and cellular processes (Aloisi et al., 2017). In recent years, genetic and molecular evidence points to PAs as essential metabolites required for tolerance to biotic and abiotic stresses. As stress-protective compounds, PAs are involved in developmental processes mediated by specific signaling pathways or in cross-regulation with other plant hormones (Alcázar et al., 2010).

There is a clear need to find solutions to help plants function under challenging environmental conditions. These solutions may include the selection of new varieties, the identification of genetic traits, and the characterization of molecules that may be helpful. The Research Topic focuses on the role of PAs in plant survival under stress conditions and includes four original research studies and one review that contribute to the understanding of the protective role of PAs in stress responses.

Many studies have reported that an increase in endogenous PAs can be beneficial for plant growth under abiotic stress conditions, suggesting that plant stress adaptation can be enhanced by increasing endogenous PAs *via* exogenous application or molecular engineering of their biosynthesis. However, such findings mainly focus on PA homeostasis and metabolism rather than PA-mediated molecular and cellular signaling cascades. In the review by Sheng et al., PA synthesis and catabolism and PAs as internal ameliorators capable of regulating stress adaptations were discussed. In particular, the authors proposed the possibility of the existence of PA-facilitated signal transduction pathways in plant tolerance to ammonium cation ( ${\text{NH}}_{4}^{\;+}$ -C) stress from a molecular and physiological perspective with respect to the recently uncovered phenomenon of urea-antagonized ${\text{NH}}_{4}^{\;+}$ -C stress. Ammonium toxicity and stress has long been recognized as one of the critical factors inhibiting the growth and productivity of many plants/crops, particularly when ${\text{NH}}_{4}^{\;+}$ -C or organic fertilizers are used or when crops and vegetables are grown in higher ${\text{NH}}_{4}^{\;+}$ -C containing hydroponic culture. This may be a more interesting topic for a deeper understanding of PA-induced growth acclimation to various stresses in future studies.

Nitrogen (N) is one of the most expensive nutrients, so improving its use efficiency is essential to reducing the cost of commercial fertilization in crop production. PAs are important N storage compounds in plants, and the ability to manipulate PAs may increase N remobilization efficiency. PA homeostasis is maintained by multiple feedback mechanisms at the levels of biosynthesis, catabolism, efflux, and uptake. The study by Stolarska et al. reported the first systematic study of PA transporters: the PA uptake transporter (PUT) and bidirectional amino acid transporter (BAT) gene families in barley. The authors identified seven PUT (*HvPUT1-7*) and six BAT *(HvBAT1-6*) genes as PA transporters in the barley genome and provided detailed characterization of these genes. Homology modeling of all studied PA transporters provided 3D structure predictions for the proteins of interest. Furthermore, molecular docking studies provided insights into the PA-binding pockets of *HvPUTs* and *HvBATs*, facilitating the understanding of the mechanisms and interactions involved in HvPUT/HvBAT-mediated transport of PAs. In addition, the authors investigated the physicochemical properties of PA transporters and discussed their function in barley development, with an emphasis on leaf senescence. These findings may lead to improved crop production through modulation of PA homeostasis.

The responses of maize to cadmium (Cd) stress were the objective of the research study by Piao et al. The photosynthetic rate, ascorbic acid-glutathione cycle system, and PA metabolism under Cd stress were investigated. The results showed that hemin could improve Cd stress tolerance. Hemin, a purified form of natural heme obtained *in vitro* by isolation and purification from animal blood, increased the content of photosynthetic pigments, the activity of photosynthetic enzymes, the total lutein cycle of leaves, and enhanced lutein cycle de-epoxidation, thus improving photosynthesis and chlorophyll fluorescence parameters of leaves under Cd stress. At the same time, hemin increased the reactive oxygen metabolic capacity and the balance of the antioxidant system in leaves and reduced the degree of membrane lipid peroxidation, thus maintaining the integrity of cell membrane structure and function under Cd stress. In addition, hemin promoted plant tolerance to Cd stress by regulating the content of PAs and enzyme activities in leaves under Cd stress. This study can provide the theoretical and experimental basis for the application of hemin for plant resilience to significantly improve the ability of plants to resist Cd stress at the seedling stage.

Winterberry (*Ilex verticillata* L. A. Gray) is a recently introduced ornamental tree species in China. Xie et al. used two-year-old cuttings of *I. verticillata* A. Gray compared with two representative cultivars (*I. verticillata* ‘Oosterwijk’ and *I. verticillata* ‘Jim Dandy’) to investigate how this plant responds to drought stress and whether exogenous spermidine (Spd) can alleviate the negative effects of drought stress. The results showed that as the level of drought stress increased, the leaves of winterberry seedlings became chlorotic and their margins became dry. Similarly, relative water content, specific leaf weight, chlorophyll content, leaf N content, net photosynthetic rate, stomatal conductance, and transpiration rate were significantly reduced, while malondialdehyde content continuously increased with the degree of drought stress. The activities of the antioxidant system enzymes increased under moderate drought stress and then decreased under severe drought stress. The levels of soluble sugars and abscisic acid continued to increase, while those of auxin and gibberellic acid decreased. Increasing the amount of exogenous Spd significantly alleviated the effects of drought stress on winterberry seedlings. The combined phenotypes and physiological indices of winterberry leaves under drought stress conditions revealed that the drought resistance of *Ilex verticillata* L. A. Gray was significantly higher than that of the other two cultivars. This finding serves as an important theoretical basis for the popularization of *I. verticillata* (L.) A. Gray.

Amine oxidases (AOs) catalyze the oxidative deamination of PAs and include two distinct classes of enzymes, copper amine oxidases (CuAOs) and flavin adenine dinucleotide (FAD)-dependent polyamine oxidases. The study by Fraudentali et al. analyzed CuAOs in *Arabidopsis*, which catalyze the oxidation of PAs to aminoaldehydes, producing hydrogen peroxide (H_2_O_2_) and ammonia. CuAOβ was reported to be involved in stomatal closure and early root protoxylem differentiation induced by the wound signal methyl jasmonate *via* apoplastic H_2_O_2_ production. Furthermore, CuAOβ is known to mediate early differentiation of the root protoxylem induced by leaf wounding, suggesting a whole-plant coordination of water supply and loss through stress-induced stomatal responses and phenotypic plasticity of the root protoxylem. In this study, the specific roles of CuAOβ and RBOHD (the D isoform of respiratory burst oxidase homolog) in the local and systemic perception of leaf and root wounding were investigated at both wounded and distal sites using *CuAOb* and *RBOHD* insertional mutants. The data show that CuAOβ-driven H_2_O_2_ production mediates both local and systemic leaf-to-leaf and root-to-leaf responses to stomatal movement, whereas RBOHD-driven H_2_O_2_ production contributes to systemic leaf-to-leaf and root-to-leaf stomatal closure. The authors hypothesized that RBOHD may act downstream of and cooperate with CuAOβ in inducing the oxidative burst that leads to systemic wound-induced stomatal closure.

Finally, the papers reviewed in this editorial highlight the link between polyamines and stress tolerance, or more broadly, plant survival. This is an exciting area of research, but many gaps must be filled before we can conclude how PAs can alleviate the effects of stressful conditions. As a first step toward developing targeted strategies to improve crop productivity and sustainability, it may be necessary to investigate the intricate mechanisms by which PAs regulate plant growth and stress tolerance. PA biosynthesis, transport, and signaling pathways, as well as interactions with other molecular players, can provide important insights into how plants cope with drought, salinity, heat, pollution, and other abiotic stresses. Furthermore, investigating the potential of genetic engineering or chemical manipulation to modulate PA levels in plants (which could act as a potential biostimulant or biocontrol agent) may provide novel approaches for improving their resilience and survival under adverse conditions. Future research on PAs has the potential to revolutionize agriculture and contribute to global food security in a rapidly changing world through interdisciplinary collaborations and advances in molecular biology, genomics, and plant physiology.

## Author contributions

All authors listed have made a substantial, direct, and intellectual contribution to the work and approved it for publication.
